# Recognition and pathological features of periampullary region adenocarcinoma with an indeterminable origin

**DOI:** 10.1002/cam4.3809

**Published:** 2021-05-19

**Authors:** Ryuji Komine, Motohiro Kojima, Genichiro Ishi, Masashi Kudo, Motokazu Sugimoto, Shin Kobayashi, Shinichiro Takahashi, Masaru Konishi, Tatsushi Kobayashi, Tetsuo Akimoto, Ayumi Murakami, Motoko Sasaki, Mariko Tanaka, Akiko Matsuzaki, Nobuyuki Ohike, Katsunori Uchida, Tomoko Sugiyama, Kenichi Hirabayashi, Takuma Tajiri, Kazuyuki Ishida, Keita Kai, Yuko Omori, Kenji Notohara, Hiroshi Yamaguchi, Yoko Matsuda, Yoshiki Naito, Yuki Fukumura, Yoshihiro Hamada, Yumi Mihara, Yohei Masugi, Naoto Gotohda, Kenichi Harada, Noriyoshi Fukushima, Toru Furukawa

**Affiliations:** ^1^ Department of Hepatobiliary and Pancreatic Surgery National Cancer Center Hospital East Chiba Japan; ^2^ Course of Advanced Clinical Research of Cancer Juntendo University Graduate School of Medicine Tokyo Japan; ^3^ Division of Pathology Exploratory Oncology Research & Clinical Trial Center National Cancer Center Hospital East Chiba Japan; ^4^ Department of Diagnostic Radiology National Cancer Center Hospital East Chiba Japan; ^5^ Department of Radiation Oncology National Cancer Center Hospital East Chiba Japan; ^6^ Department of Pathology Saiseikai Yokohamashi Nanbu Hospital Kanagawa Japan; ^7^ Department of Human Pathology Kanazawa University Graduate School of Medical Sciences Kanazawa Japan; ^8^ Department of Pathology University of Tokyo Tokyo Japan; ^9^ Department of Pathology University of the Ryukyus Okinawa Japan; ^10^ Division of Pathology Shizuoka Cancer Center Shizuoka Japan; ^11^ Department of Pathology Mie University Mie Japan; ^12^ Department of Pathology Tokai University School of Medicine Kanagawa Japan; ^13^ Department of Diagnostic Pathology Dokkyo Medical University Tochigi Japan; ^14^ Department of Pathology Saga University Hospital Saga Japan; ^15^ Department of Investigative Pathology Tohoku University Graduate School of Medicine Sendai Miyagi Japan; ^16^ Department of Pathology Kurashiki Central Hospital Okayama Japan; ^17^ Department of Pathology Saitama Medical University Saitama Japan; ^18^ Department of Pathology Kagawa University Kagawa Japan; ^19^ Department of Diagnostic Pathology Kurume University Hospital Fukuoka Japan; ^20^ Department of Human Pathology School of Medicine Juntendo University Tokyo Japan; ^21^ Department of Pathology Fukuoka University Fukuoka Japan; ^22^ Department of Pathology National Hospital Organization Nagasaki Medical Center Hospital Nagasaki Japan; ^23^ Department of Pathology Keio University School of Medicine Tokyo Japan; ^24^ Department of Pathology Jichi Medical University Tochigi Japan

**Keywords:** ampulla of Vater carcinoma, distal bile duct carcinoma, indeterminable tumor primary, pancreatic ductal adenocarcinoma, periampullary region

## Abstract

Determination of the primary tumor in periampullary region carcinomas can be difficult, and the pathological assessment and clinicopathological characteristics remain elusive. In this study, we investigated the current recognition and practices for periampullary region adenocarcinoma with an indeterminable origin among expert pathologists through a cognitive survey. Simultaneously, we analyzed a prospective collection of cases with an indeterminable primary tumor diagnosed from 2008 to 2018 to elucidate their clinicopathological features. All cases with pathological indeterminable primary tumors were reported and discussed in a clinicopathological conference to elucidate if it was possible to distinguish the primary tumor clinically and pathologically. From the cognitive survey, over 85% of the pathologists had experienced cases with indeterminable primary tumors; however, 70% of the cases was reported as pancreatic cancer without definitive grounds. Interpretation of the main tumor mass varied, and no standardized method was developed to determine the primary tumor. During a prospective study, 42 of the 392 periampullary carcinoma cases (10.7%) were considered as tumors with a pathological indeterminable origin. After the clinicopathological conferences, 21 (5.4%) remained indeterminable and were considered final indeterminable cases. Histological studies showed that the tumors spread along both the bile duct and main pancreatic duct; this was the most representative finding of the final indeterminable cases. This study is the first to elucidate and recognize the current clinicopathological features of periampullary region adenocarcinomas with an indeterminable origin. Adequate assessment of primary tumors in periampullary region carcinomas will help to optimize epidemiological data of pancreatic and bile duct cancer.

## Graphical abstract

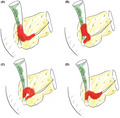

## 1 INTRODUCTION

Theoretically, clinicopathological and biological features of carcinomas are highly dependent on their primary structure. Therefore, distal bile duct carcinoma (DBDC), ampulla of Vater carcinoma (AmpC), and pancreatic ductal adenocarcinoma (PDAC) are classified in separate chapters in the World Health Organization (WHO) classification and are staged according to the distinct American Joint Committee of Cancer (AJCC)/Union for International Cancer Control‐TNM staging guidelines. Accordingly, they are treated differently based on the respective National Comprehensive Cancer Network guidelines (NCCN).^1^, ^2^ Epidemiologically, PDAC is the fourth leading cause of cancer deaths in Japan, as well as in Europe and the United States, with an overall 5‐year survival rate of only about 7%.^3^, ^4^, ^5^ In addition, biliary tract cancer, including DBDC and AmpC, is the sixth leading cause of death in Japan, and its incidence has been increasing worldwide over the last few decades, particularly in East Asia^5^; however, it is regarded as a relatively rare disease in Western countries.^5^, ^6^, ^7^

The periampullary region is a complex area composed of three histologically and physiologically distinct anatomic structures, namely, the common bile duct (CBD), pancreatic duct, and ampulla of Vater (AoV). However, the many structures of the pancreatobiliary system share common developmental processes and many biological features. Specific features or biomarkers to determine DBDC, PDAC, and AmpC are very limited,^8^ and thus, determining the primary origin in a carcinoma arising in this narrow area can be difficult. The current WHO classification defines biliary tract cancer by the location of the primary tumor mass only, though the AJCC 8th edition states that identifying the tumor origin in the periampullary region can be difficult due to the intimate association of the bile duct with the pancreas and their similar immunophenotype.^9^ In fact, scientific information about the discrimination of primary tumors in such cases is lacking. Furthermore, the term “the location of main tumor mass” in the WHO classification may bring various interpretations. For example, because the biliary tract is much smaller than the pancreas, if only the invasive tumor area was regarded as the primary tumor site's determinant, many tumors originating from the bile duct would be classified as PDAC in the setting of massive invasion into the pancreas. The tumor origin may also be presumed based on many other features without hierarchy. For instance, intraductal tumor spread or tumor spread along the organ structure may also represent the primary site, and radiological findings can be used to estimate the primary tumor location. These findings are not always consistent, and the hierarchy of these findings for determining the primary tumor has not been determined. Due to these complexities, determination of the primary tumor origin for periampullary region carcinomas is not standardized, and inconsistent assessments and reporting of primary tumor locations may have occurred in routine practice. To solve this problem and to establish the baseline for a standardized assessment of the primary tumor site for periampullary region carcinomas, two matters should be clarified. One is to comprehend the current pathological assessment and recognition of periampullary region adenocarcinomas with an indeterminable origin (PRAIO). The other is to clarify the clinicopathological features of these tumors.

In this study, we first conducted a cognitive survey to evaluate the current pathological assessment and recognition of PRAIO among expert pathologists to determine whether the concept of PRAIO could be shared and whether the identification method for primary tumors’ sites was identical among pathologists. Second, we prospectively collected PRAIO cases according to a consistent selection method and investigated their clinicopathological features to elucidate pathological features which may hinder the determination of the primary tumor. Together with these results, we tried to provide scientific information and clues for standardized reporting of the primary tumors of PRIAO.

## 2 MATERIALS AND METHODS

### 2.1 Cognitive survey using a questionnaire

First, we conducted a cognitive survey using a questionnaire to elucidate the current assessment and recognition of PRAIO cases (Table S1). This survey was sent to expert pathologists belonging to the Pancreatobiliary Pathology Club Japan through e‐mail from May 2019 to December 2019. The questionnaire consisted of the following: a profile of the pathologist (Q1), recognition of the existence and the estimated frequency of PRAIO (Q2), important findings in determining the primary tumor site (Q3, 4), an experience of cases in which a pathological diagnosis of the tumor's primary origin did not match the clinical diagnosis (Q5), and how to determine the location of the primary tumor (Q6). Although the determination of the primary tumor location is essential in characterizing the tumor's primary origin in the WHO classification, pathological findings to assess the primary tumor were not discussed among the pathologists. For this reason, we specifically asked for the reasons for reporting the location and if the tumor involved both the pancreas and bile duct in equal proportion (Q7). Finally, we asked why pathologists found it difficult to diagnose periampullary region adenocarcinomas (Q8) and whether histological evaluation alone had limitations for determining the primary tumor (Q9).

### 2.2 Case selection

We prospectively selected PRAIO in pancreatoduodenectomy specimens retrieved from the National Cancer Center Hospital East, Japan, from January 2008 to December 2018. The study conforms to the provisions of the Declaration of Helsinki and was approved by the hospital ethics committee (approval number: 2017–483). Since the definition of PRAIO was not established, the case selection was performed by a stylized evaluation process using a multiple modality approach as follows. First, we collected information on all tumors located in the periampullary region, specifically those for which the organ of the primary tumor could not be determined by pathological findings alone (e.g., tumor involved the pancreas and the bile duct evenly); we reported these tumors as pathological PRAIO (pPRAIO). In this group, we also included the cases with tumors identified as “more likely PDAC (AmpC or DBDC), though not definitive.” Pathological findings were independently evaluated by two or more expert pathologists who specialized in hepatobiliary‐pancreas in our hospital. All pPRAIO were reported as indeterminable cases of either PDAC or AmpC (PRAIO‐PA), either PDAC or DBDC (PRAIO‐PB), and either AmpC or DBDC (PRAIO‐AB). All pPRAIO were discussed at the clinicopathological conferences in which expert pathologists, radiologists, ultrasonography technologists, hepatobiliary pancreatic physicians, and surgeons participated. The final diagnosis was assigned as per the consensus that emerged at the conference. At the conference, the pathologists presented the mapping of the histological invasion area on a photograph of the tumor's cut surface, and histological features that helped determine the primary tumor site were discussed. The participants discussed the chronological direction of tumor infiltration by matching the pathological findings and clinical information such as initial symptoms, preoperative blood test results, or others. We then reverified the primary tumor mass location in preoperative images using multiple modalities (abdominal ultrasonography, computed tomography [CT], magnetic resonance imaging, and endoscopic retrograde cholangiopancreatography [ERCP]). With focus on consistency with preoperative imaging diagnosis, cases in which the primary tumor site could not be determined by pathological or clinical evaluation were defined as final PRAIO (fPRAIO).

### 2.3 Histological evaluation

According to the seventh edition of the General Rules for the Study of Pancreatic Cancer by the Japan Pancreas Society and the sixth edition of the General Rules for Clinical and Pathological Studies on Cancer of the Biliary Tract by the Japan Biliary Association, the duodenum was opened longitudinally on the opposite side of the pancreatic head, and the whole specimens were fixed in 10% buffered formalin for 24 to 48 h. Subsequently, the tissue specimen was cut into 5‐mm thick slices in a plane perpendicular to the duodenal axis to investigate 7 to 10 cut surfaces with pancreatic fields. The main pancreatic duct was not opened, but the CBD was opened in the longitudinal direction on the posterior side in the cases clinically diagnosed as DBDC or AmpC. Two experienced surgical pathologists (MK and GI) and members of the hepatobiliary and pancreatic field (RK and others) reviewed the specimens to confirm the pathological findings and then created a mapping of the tumor area on each plane. In this study, we evaluated eight pathological features for the determination of the primary tumor based on previous reports.^10^, ^11^, ^12^, ^13^, ^14^, ^15^, ^16^, ^17^, ^18^, ^19^ (Figure S1). These findings were as follows: From the maximum tumor plane, we assessed the following four items: (1) maximum tumor size for the axial direction, (2) maximum tumor size for the sagittal direction, (3) presence or absence of tumor involvement of the bile duct surface, and (4) presence or absence of symmetric and/or circumferential involvement of the bile duct. Next, from the tumor margin or non‐infiltrative plane, the presence or absence of a high‐grade intraepithelial lesion, including carcinoma in situ or cancerization (5) of the bile duct and (6) of the main pancreatic duct were recorded. From the whole‐tumor mapping, (7) the presence or absence of tumor progression along the long axis of the bile duct wall, and (8) the presence or absence of tumor progression along the long axis of the main pancreatic duct were assessed. The last two were judged to be positive when there was tumor infiltration or high‐grade epithelial lesions in the bile duct wall (or around the main pancreatic duct) on three or more continuous sections. Continuous variables were measured digitally using the NDP view 2 application after scanning the maximal infiltrated section of the tumor using NanoZoomer (HAMAMATSU PHOTONICS Corporation, Hamamatsu, Japan).

### 2.4 Evaluation of clinicopathological parameters

Demographic and clinicopathological data included age, sex, body mass index (BMI), and preoperative serum levels of bilirubin, albumin, and carbohydrate antigen (CA) 19–9. Preoperative laboratory data were extracted from the chart review. Follow‐up information was obtained through medical chart review.

### 2.5 Statistical analysis

The descriptive variables are expressed in mean (standard deviation [SD]), median (range or interquartile range [IQR]), or frequency count (%) for continuous and categorical variables, as appropriate.

The differences in the eight pathological variables between fPRAIO, PDAC, AmpC, and DBDC were determined using chi‐squared test or Fisher's exact test for categorical variables and the Mann–Whitney test for continuous variables. Overall survival (OS) and recurrence‐free survival (RFS) were calculated by creating Kaplan–Meier curves, and differences in survival between fPRAIO, PDAC, AmpC, and DBDC were assessed using the log‐rank test. *p*‐values <0.05 were considered statistically significant. To analyze differences in the OS and RFS between the groups, a multiple comparison procedure using the Bonferroni test was also performed, with a *p*‐value <0.0083 considered statistically significant. All statistical analyses were carried out using JMP version 14 statistical software (SAS Institute, Cary, NC, USA).

## 3 RESULTS

### 3.1 Results of cognitive survey

Twenty‐one expert pathologists from 18 hospitals (15 universities and 3 central hospitals) responded to the questionnaire. The questionnaire response rate was 15%.

**FIGURE 1:**
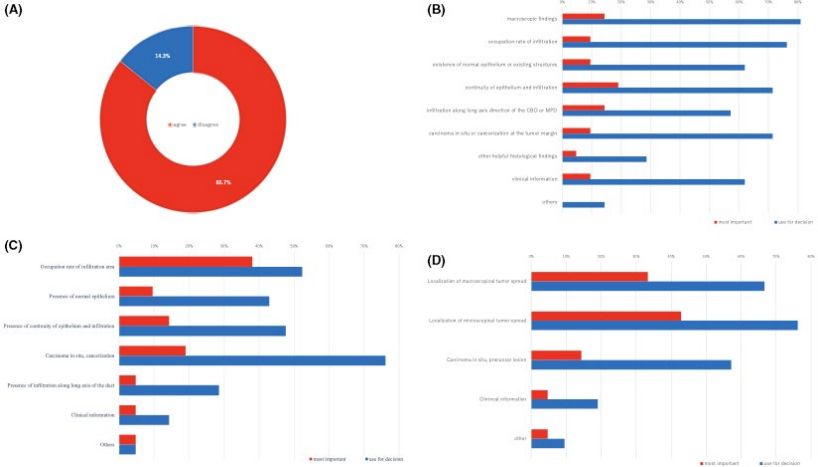
Results of cognitive survey. (A) Consent to the existence of PRAIO cases. A total of 85.7% of the participating pathologists agreed to the existence of PRAIO cases. (B) Clinicopathological findings for identification of tumor origin. All participants answered that they assessed the primary tumor site with multiple findings, but the findings were weighted differently. (C) Clinicopathological findings of the primary tumor location. (D) Similar question to (C) in a hypothetical case where the tumor would equally involve the bile duct and the pancreas macroscopically and microscopically. Interpretation of the location of the primary tumor mass that is described as specifying the primary tumor site in the WHO classification, was assessed through multiple findings by all the participants. The weightage of the findings also varied. This was similar in a hypothetical case where gross and histological tumor spread were equal. PRAIO: Periampullary region adenocarcinoma with indeterminable origin, WHO: World Health Organization.

The average professional experience of the respondents was 17.8 years. Of these, 85.7% (18/21) agreed to the existence of the PRAIO (Figure 1A). Regarding estimated frequency, the most common response among the pathologists who agreed to the existence of PRAIO was 1 PRAIO for 50 to 100 cases (66.7% (12/18) answered). Of these pathologists, 72.2% (13/18) answered that they ultimately diagnosed PRAIO cases as PDAC without a definitive basis, and only 16.7% (3/18) reported that PRAIO could be diagnosed as PDAC and BTC. Additionally, 90.5% (19/21) of the respondents agreed that histology alone can limit the determination of a tumor's origin.

From the responses on how to determine the tumor's primary origin (Q3 and 4), it was found that all respondents assessed the primary tumor's origin based on multiple findings. In addition, the weightage of these findings had huge variations. For instance, over 80% (17/21) of the respondents answered that macroscopic findings were helpful; however, only 14.2% (3/21) considered them as the most important finding. Further, there was no consensus reached for the most important finding among respondents (Figure 1B). Similarly, there was a lack of standardized interpretation of the primary tumor mass location (Q6 and 7). Of the respondents, 90.5% (19/21) assessed the mass with multiple findings, and none of the findings were considered the most important by more than 50% of the respondents (Figure 1C). These results indicated that the interpretation of the location of the primary tumor was not uniform. In addition, for the hypothetical question of when the tumors infiltrated both structures evenly in gross and microscopic appearance, the choice of the most important finding by the participants varied significantly (Figure 1D).

### 3.2 Frequency of PRAIO

**FIGURE 2:**
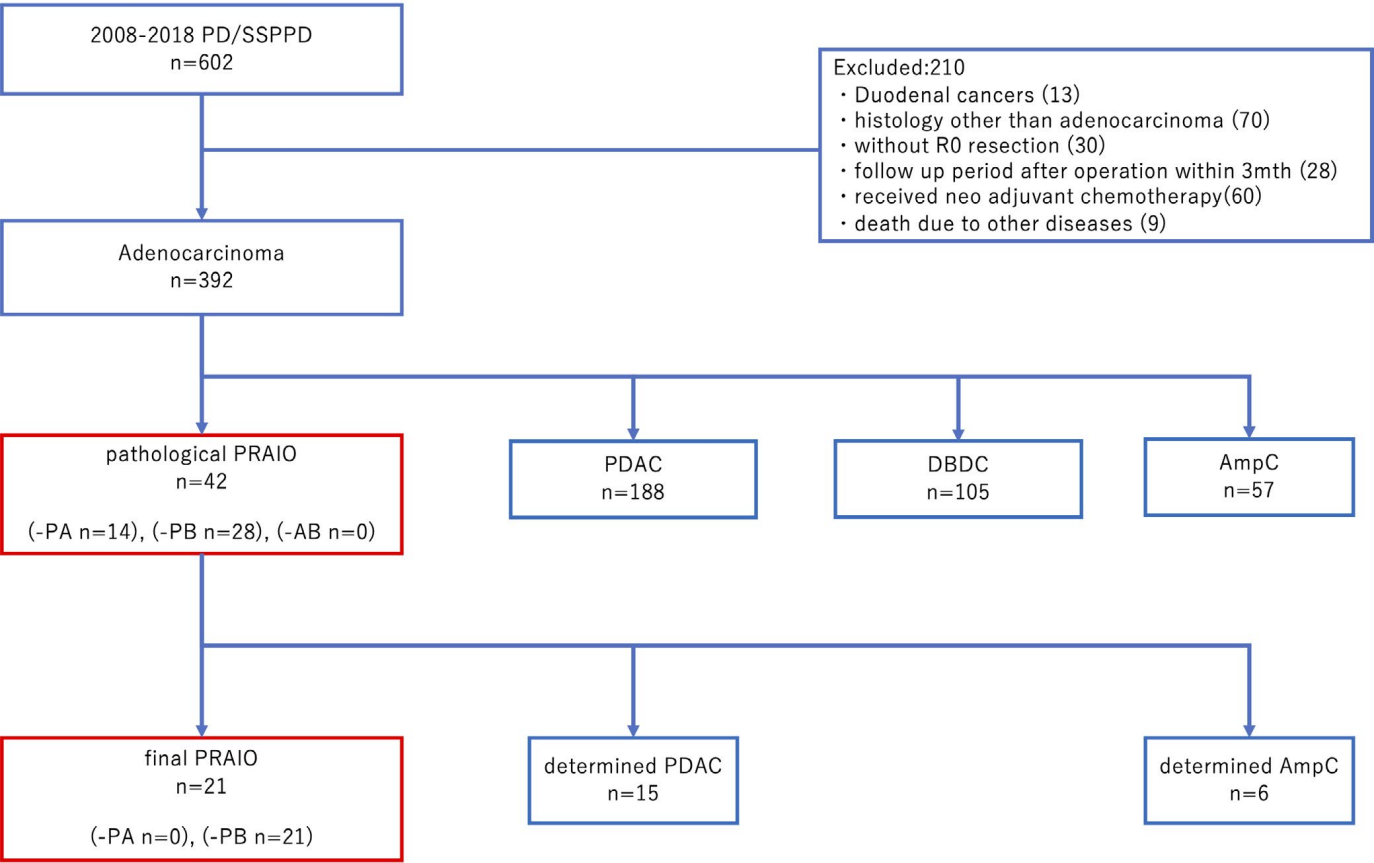
Case selection chart. Of 392 cases that met the inclusion criteria, 42 cases were diagnosed as pathological indeterminable cases (pathological PRAIO) through assessment by two or more expert pathologists. All the pathological PRAIO cases were reported as either indeterminable PDAC or AmpC (pPRAIO‐PA), and either PDAC or DBDC (pPRAIO‐PB), and discussed in the clinicopathological conference. Their primary tumor sites were distinguished after considering consistency with diagnostic imaging. After that, 21 cases were indeterminable and classified separately as final PRAIO. AmpC; ampulla of Vater carcinoma, DBDC; distal bile duct carcinoma, PDAC; pancreatic ductal adenocarcinoma, PD; Pancreaticoduodenectomy, PRAIO: Periampullary region adenocarcinoma with indeterminable origin, SSPPD: subtotal stomach‐preserving pancreaticoduodenectomy.

During the study period, 602 pancreatoduodenectomy specimens were retrieved. The following cases were excluded: (1) duodenal cancers (13 cases), (2) histology other than adenocarcinoma (70 cases), (3) specimens without an R0 resection (30 cases), (4) short follow‐up period within 3 months after operation (28 cases), (5) received neoadjuvant chemotherapy (60 cases), and (6) death due to other diseases (9 cases). In the remaining 392 cases, 42 cases (10.9%) were assessed as pPRAIO cases. These 42 pPRAIO were consisted of 14 pPRAIO‐PA and 28 pPRAIO‐PB cases, while no pPRAIO‐AB case was recorded. No pancreatobiliary maljunction was seen among the 42 pPRAIO cases. Through the discussion in the conference, the primary tumor's location was determined in 21 cases, with a consensus reached among all participants who attended the conference. In addition, 14 pPRAIO‐PA were diagnosed as 4 AmpC and 10 PDAC, and the 7 pPRAIO‐PB were diagnosed as 2 DBDC and 5 PDAC; however, 21 pPRAIO‐PB cases remained indeterminate and were assessed as fPRAIO (Figure 2). All fPRAIO were assessed from pPRAIO‐PB, and the frequency of fPRAIO was 5.4% in periampullary region carcinoma.

### 3.3 Histological and clinicopathological characteristics of fPRAIO

Table 1 shows the univariate analysis of eight histological features of fPRAIO compared to those of PDAC, AmpC, and DBDC. The tumor sizes of fPRAIO were similar to those of PDAC and were significantly larger than those of AmpC and DBDC. However, fPRAIO frequently involved the bile duct surface and showed symmetric and/or circumferential involvement of the bile duct. In addition, fPRAIO frequently showed high‐grade intraepithelial lesions in both the bile duct and main pancreatic duct and spread along their long axes. Of note, tumor involvement of the bile duct surface and the longitudinal spread along both the bile duct and main pancreatic duct were the most common features found in more than 95% of fPRAIO cases.

**TABLE 1:** Univariate analysis of eight pathological variables showing differences between fPRAIO and PDAC / AmpC / DBDC.

	fPRAIO (*n* = 21)	PDAC (*n* = 203)	AmpC (*n* = 63)	DBDC (*n* = 105)	*P*‐value		
					vs PDAC	vs AmpC	vs DBDC
(1) Maximum tumor length toward the axial direction, mm, mean	21.6 ± 6.2	22.1 ± 7.7	7.1 ± 5.9	5.7 ± 3.9	0.8358	<0.0001	<0.0001
(2) Maximum tumor length toward the sagittal direction, mm, mean	23.6 ± 7.9	25.6 ± 9.7	3.1 ± 7.1	0.9 ± 2.5	0.3862	<0.0001	<0.0001
(3) Tumor involvement of the bile duct surface, *n* (%)	21 (100)	38 (18.7)	63(100)	105 (100)	<0.0001		
(4) Presence of symmetric/circumferential involvement of the bile duct, *n* (%)	16 (76.2)	60 (29.6)	29 (46.0)	56 (53.3)	<0.0001	0.0226	0.0582
(5) High‐grade intraepithelial lesion of the bile duct, *n* (%)	13 (61.9)	0	20 (31.8)	23 (21.9)	<0.0001	0.0202	0.0009
(6) High‐grade intraepithelial lesion of the main pancreatic duct, *n* (%)	10 (47.6)	50 (24.6)	10 (15.9)	3 (2.9)	0.0360	0.0064	<0.0001
(7) Presence of tumor progression along the long axis of the bile duct, *n* (%)	20 (95.2)	1 (0.5)	26 (41.3)	80 (76.2)	<0.0001	<0.0001	0.0728
(8) Presence of tumor progression along the long axis of the main pancreatic duct, *n* (%)	20 (95.2)	154 (75.9)	4 (6.4)	0	0.0517	<0.0001	<0.0001

Abbreviations: AmpC; ampulla of Vater carcinoma, DBDC; distal bile duct carcinoma, PDAC; final periampullary region adenocarcinoma with indeterminable origin; pancreatic ductal adenocarcinoma, PRAIO.

pPRAIO‐PA cases had larger tumor diameters than those observed with AmpC; furthermore, more than 70% of them involved the bile duct surface and infiltrated along the long axis of the main pancreatic duct. However, they did not frequently spread along the long axis of the bile duct (Table 2). Together with the radiological findings, the pPRAIO‐PA were determined to be six AmpC and eight PDAC in conferences. In four pPRAIO‐PA, a well‐defined mass protruding into the duodenal lumen was confirmed by ERCP and CT and diagnosed as AmpC. Eight pPRAIO‐PA cases were determined to be PDAC due to mass formation with gradual enhancement confined to the head of the pancreas on preoperative CT. Although the remaining two pPRAIO‐PA cases had no obvious mass formation, they were determined to be AmpC due to the CBD dilation, obstruction at the AoV, and thickening of the bile duct wall with enhancement.

**TABLE 2:** Univariate analysis of eight pathological variables between pPRAIO‐PA and PDAC, pPRAIO‐PA and AmpC.

	pPRAIO‐PA (*n* = 14)	PDAC (*n *= 188)	AmpC (*n* = 57)	‐value	
				PDAC	AmpC
(1) Maximum tumor length toward axial direction, mm, mean	21.2 ± 7.5	22.1 ± 7.8	5.9 ± 4.5	0.8475	<0.0001
(2) Maximum tumor length toward sagittal direction, mm, mean	21.6 ± 13.9	25.7 ± 9.6	1.8 ± 3.7	0.2718	<0.0001
(3) Tumor involvement of the bile duct surface, *n* (%)	13 (92.8)	26 (13.8)	57 (100)	<0.0001	0.1972
(4) Presence of symmetric/circumferential involvement of the bile duct, *n* (%)	8 (57.1)	52 (27.7)	28 (49.1)	0.0310	0.7668
(5) High‐grade intraepithelial lesion of the bile duct, n (%)	2 (14.3)	0	18 (31.6)	0.0045	0.3213
(6) High‐grade intraepithelial lesion in the main pancreatic duct, *n* (%)	8 (57.1)	42 (22.3)	6 (10.5)	0.0074	0.0005
(7) Presence of tumor progression along the long axis of the bile duct, *n* (%)	4 (28.6)	0	21 (36.8)	<0.0001	0.7568
(8) Presence of tumor progression along the long axis of the main pancreatic duct, *n* (%)	11 (78.6)	142 (75.5)	0	1.0000	<0.0001

Abbreviations: AmpC; ampulla of Vater carcinoma, PDAC; pancreatic ductal adenocarcinoma, pPRAIO‐PA; pathological periampullary region adenocarcinoma with indeterminable origin either pancreatic ductal adenocarcinoma or ampulla of Vater carcinoma.

**FIGURE 3:**
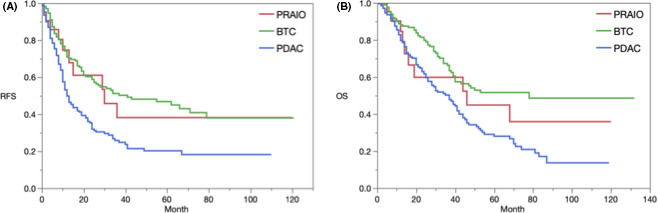
Results of survival analysis. (A) RFS. The Median follow‐up was 25.0 months (IQR: 30.2 to 35.4 months). The median RFS for all the cases was 15 months (95% confidence interval (CI) 12 to 17 months). The 1/3/5‐year recurrence‐free rates for PRAIO, PDAC, AmpC, and DBDC were 74.6/38.2/38.2, 49.8/25.8/20.2, 78.8/55.4/51.9, and 66.4/47.3/44.1 (log‐rank *p* < 0.0001). With the Bonferroni test for multiple comparison (*p*‐value <0.0083 was set as significant), PRAIO versus AmpC (*p* = 0.3032) and versus DBDC (*p* = 0.9275) were not different. (B) OS. The median OS for all the cases was 25.0 months (95% CI 22 to 28 months). The 1/3/5‐year survival rates for PRAIO, PDAC, AmpC, and DBDC were =84.6/59.8/44.8, 79.5/50.1/28.0, 93.1/75.4/52.7, and 85.2/61.5/50.9 (log‐rank *p* < 0.0001). The OS of PRAIO was halfway between PDAC and AmpC/DBDC. With the Bonferroni test, PRAIO versus PDAC (*p* = 0.6239), PRAIO versus AmpC (*p* = 0.0898), and PRAIO versus DBDC (*p* = 0.3777) were not significantly different. AmpC; ampulla of Vater carcinoma, DBDC; distal bile duct carcinoma, PDAC: pancreatic ductal adenocarcinoma, PRAIO; Periampullary region adenocarcinoma with indeterminable origin, RFS: recurrence‐free survival, IQR: interquartile range, OS: overall survival.

Table 3 shows that there was no significant difference in patient background between fPRAIO, PDAC, AmpC, and DBDC. The rates of lymph node metastasis in PRAIO were similar to those in PDAC, but significantly higher than those in AmpC and DBDC. The RFS was significantly different between PRAIO, PDAC, AmpC, and DBDC (log‐rank *p* < 0.0001), and the RFS associated with PRAIO was close to that for AmpC (*p* = 0.3032) and DBDC (*p* = 0.9275). OS was significantly different in these four groups (log‐rank *p* = 0.0001). Although there was no significant difference found with the Bonferroni test, PRAIO had an intermediate OS between PDAC (*p* = 0.6239) and AmpC (*p* = 0.0898)/DBDC (*p* = 0.3777; Figure 3).

**TABLE 3:** Clinicopathological characteristics of PRAIO, PDAC, AmpC, and DBDC.

	PRAIO (*n* = 21)	PDAC (*n* = 203)	AmpC (*n* = 63)	DBDC (*n* = 105)	*p*‐value		
					vs PDAC	vs AmpC	vs DBDC
Age, year	69.9 ± 5.4	68.0 ± 10.3	70.3 ± 10.2	70.9 ± 8.3	0.7934	0.3434	0.3434
Sex, male, *n* (%)	15 (71.4)	123 (60.6)	35 (55.7)	79 (75.2)	0.6455	0.4485	0.4236
BMI	20.9 ± 2.9	20.7 ± 3.1	21.4 ± 3.4	21.7 ± 2.9	0.5359	0.8283	0.5127
Past illness							
HT, *n* (%)	10 (47.6)	82 (42.9)	25 (39.7)	36 (34.3)	0.8200	0.8031	0.4643
DM, *n* (%)	5 (23.8)	50 (24.6)	9 (14.3)	24 (22.9)	1.0000	0.3244	1.0000
Laboratory data							
Alb, g/dL	4.2 [3–4.6]	4.2 [2.1–5.0]	4 [2.6–5.1]	3.9 [2.7–4.7]	0.6344	0.6293	0.1783
T‐Bil, g/dL	3.0 [4–21.0]	1.4 [0.2–31.9]	0.79 [0.3–15.9]	3.1 [0.2–40.1]	0.1163	0.0039	0.6872
CEA, ng/mL	2.7 [1.0–6.0]	3.0 [0.2–27.7]	2.2 [0.6–11.3]	2.3 [0.2–10.6]	0.3189	0.2987	0.5424
CA19‐9, U/mL	37.9 [12.7–319]	73.4	20.6 [0.6–975]	47.7 [0.3–18020]	0.1942	0.0059	0.7742
Pathological results							
Histological type, pap‐tub, n (%)	19 (90.5)	181 (89.2)	60 (95.2)	149 (88.7)	1.0000	0.5948	1.0000
LI positive, *n* (%)	19 (90.5)	161 (79.3)	40 (63.5)	72 (68.6)	0.3843	0.0261	0.0593
VI positive, *n* (%)	18 (85.7)	179 (88.2)	26 (41.3)	65 (61.9)	0.3099	0.0022	0.1325
NI positive, *n* (%)	17 (80.9)	186 (91.6)	18 (28.6)	86 (81.9)	0.1179	<0.0001	1.0000
Lymph meta positive, *n* (%)	16 (76.2)	140 (68.9)	29 (46)	47 (44.8)	0.6216	0.0226	0.0153
Recurrence, *n* (%)	11 (52.4)	139 (68.8)	24 (38.1)	57 (54.3)	0.1455	0.4543	0.6364
Site of recurrence							
Liver, *n* (%)	5 (23.8)	55 (27.2)	10 (15.9)	28 (26.7)	1.0000	0.7419	0.5886
Lung, *n* (%)	0	18 (8.9)	5 (7.9)	6 (5.7)	0.2304	0.3247	0.5883
Local, *n* (%)	5 (23.8)	45 (22.3)	6 (9.5)	14 (13.3)	0.7905	0.1323	0.3121
Dissemination, *n* (%)	0	18 (8.9)	3 (4.8)	10 (9.5)	0.2304	0.5696	0.2114

Abbreviations: AmpC; ampulla of Vater carcinoma, BMI; body mass index, CA19‐9; carbohydrate antigen 19–9, CEA; carcinoembryonic antigen, DBDC; diabetes mellitus, HT; distal bile duct carcinoma, DM; final periampullary region adenocarcinoma with indeterminable origin, VI; hypertension, LI; lymphatic invasion, NI; neural invasion, PDAC; pancreatic ductal adenocarcinoma, PRAIO; vascular invasion.

### 3.4 Subclassification of fPRAIO

**FIGURE 4:**
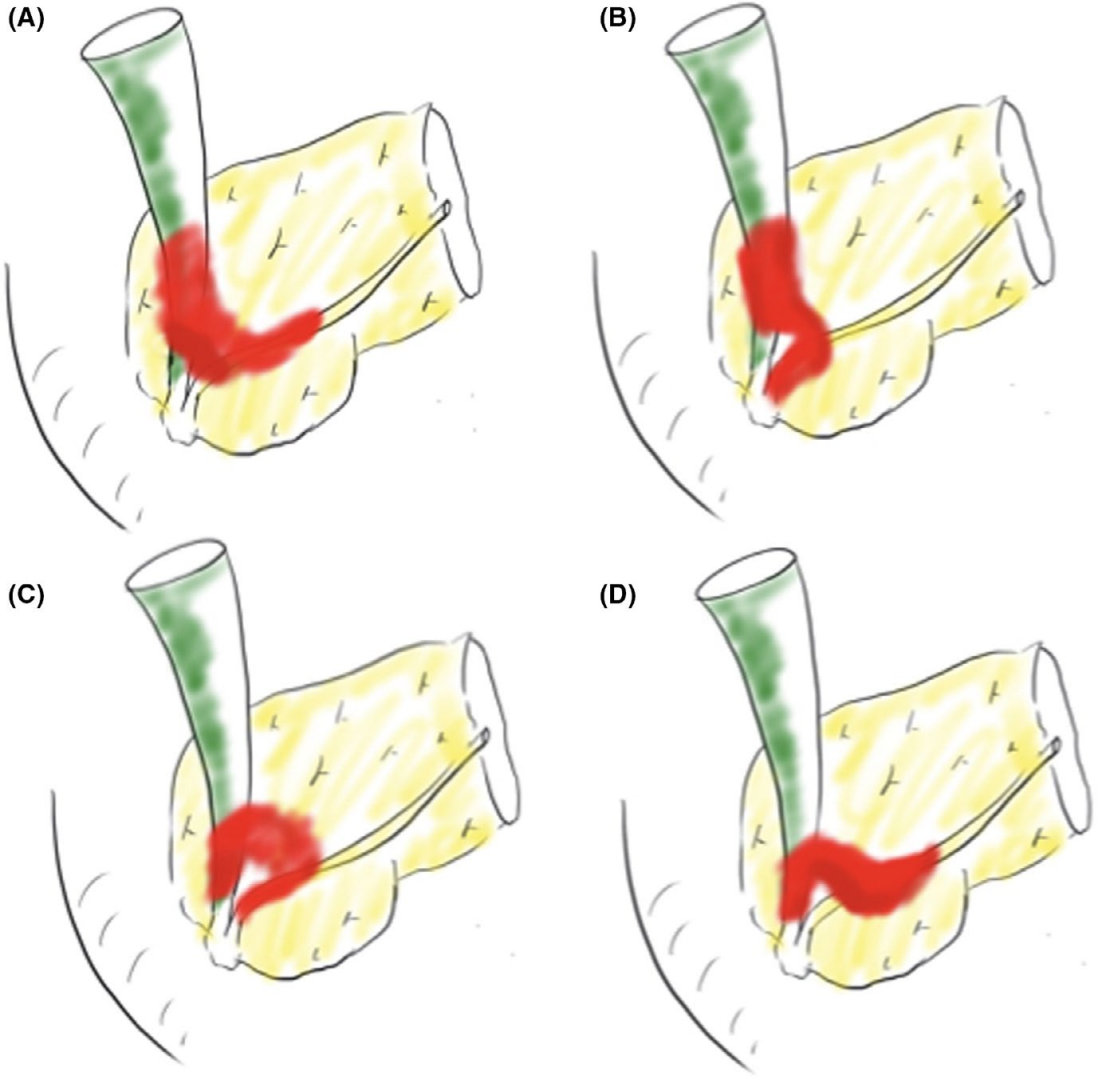
Schema of subclassification of PRAIO. PRAIO could be subclassified into the following five types according to the direction of tumor infiltration along the long axis of the bile duct or pancreatic duct. From the maximal section of the tumor, it was considered as central type if the longitudinal invasion was in the AoV or duodenal direction and peripheral type if in the contralateral direction. (A) Peripheral‐Peripheral type: 9.5% (2/21), (B) Peripheral‐Central type: 28.6% (6/21), (B) Central‐Central type: 28.6% (6/21), (D) Central‐Peripheral type: 28.6% (6/21), and other type: 9.5% (2/21). AoV: ampulla of Vater, PRAIO: Periampullary region adenocarcinoma with indeterminable origin.

Based on the morphological information, we were able to subclassify fPRAIO into the following types according to the direction of tumor spread along the long axis of the bile duct and main pancreatic duct. Tumor spread toward the AoV or duodenum was defined as the central type, and that toward the contralateral side were defined as the peripheral type (Figure 4). Of the 21 fPRAIO cases, 2 (9.5%) were the peripheral‐peripheral type in which the tumor spread toward the periphery of the bile duct wall and main pancreatic duct (Figure 4A), 6 cases (28.6%) were the peripheral‐central type in which the tumor spread toward the periphery of the bile duct wall and central side of the pancreatic duct (Figure 4B), 5 cases (23.8%) were the central‐central type in which the tumor spread toward the central sides of the bile duct and the pancreatic duct (Figure 4C), and 6 cases (28.6%) were the central‐peripheral type in which the tumor spread toward the central side of the bile duct and toward the peripheral side of the pancreatic duct (Figure 4D). The other two cases (9.5%) did not match the above four types; for example, the tumor infiltrated the central and peripheral direction of both ducts, like a dumbbell.

## 4 DISCUSSION

Recent progress in cancer therapy has led to the establishment of guidelines based on the primary tumor site. Although therapeutic strategies of DBDC, PDAC, and AmpC are partly similar, advances in adjuvant chemotherapy require a strict distinction between these carcinomas. For PDAC, multi‐agent chemotherapy such as modified FOLFIRINOX has become a standard; however, a standard adjuvant therapy for biliary tract cancer remains unclear.^20^, ^21^, ^22^ Therefore, determination of the primary tumor site in either PDAC or DBDC/AmpC in a standardized manner is becoming more important in patient management. Because the periampullary region is composed of several organs packed in close proximity, there may be some cases in which it is difficult to determine the primary tumor site. Although this phenomenon is described in the NCCN guidelines and WHO classification, the existing studies on PRAIO is limited due to the complexity of its delineation. Our study clarified for the first time that variable features were randomly adopted to discriminate primary tumors among pathologists. However, the existence of indeterminable cases defined as PRAIO were recognized by over 85% of expert pathologists in the hepatobiliary‐pancreatic field. This survey also revealed the complexity in determining the primary tumor site and primary tumor mass by conventional pathological examinations alone. Many pathological items without hierarchy for the determination seemed to be missing from a consistent assessment of the primary tumor site. Similarly, a lack of clear criteria for assessing the primary tumor mass location may also impede consistency in the assessment of primary tumor sites. In addition, most of these pathological PRAIO cases were identified as PDAC in many institutions without a clear basis. These results suggest that PRAIO may influence the construction of a standardized cancer data set.

Through our prospective selection strategy, clinicopathological features of fPRAIO were successfully elucidated. By this selection method that ensured the objectivity from multimodality, we could elucidate basic pathological and clinicopathological features of fPRAIO for the first time.

Initially, we found a substantial frequency of pPRAIO, and 10.2% of the 392 periampullary region adenocarcinoma was regarded as pathologically indeterminable, and 5.4% were assessed as fPRAIO. This frequency also suggests that PRAIO may affect the construction of standardized pathological assessments, and thus, clinical management of PRAIO should be discussed urgently. Of the included pPRAIO, we found many pPRAIO‐BP and few pPRAIO‐PA. On the contrary, no pPRAIO‐AB case was recorded in this study, and all fPRAIO cases were pPRAIO‐BP. Further pathological investigation regarding pPRAIO‐PA and ‐AB are necessary to elucidate novel subgroups of periampullary adenocarcinoma. Anyway, this study revealed that discrimination of the primary tumor between PDAC and DBDC would be difficult and very important in clinicopathological practice. The clinicopathological analysis of fPRAIO also revealed unique morphological characters that were distinct from typical PDAC, AmpC, and DBDC. The representative feature of fPRAIO was the simultaneous tumor spread along the long axis of the bile duct and main pancreatic duct, and the tumor involvement of the bile duct surface. The tumor spread pattern in the bile duct and main pancreatic duct was variable. These findings should be the factor that disturb pathological determination of the primary tumor. In addition, fPRAIO were significantly different from AmpC and DBDC and more similar to PDAC in terms of maximum tumor diameter and rate of lymph node metastasis. Nevertheless, their prognosis was relatively similar to that of AmpC and DBDC. This paradoxical result suggests that fPRAIO itself may have unique properties. Schumuck et al^23^ opted to analyze tumors arising from the pancreaticobiliary junction area separately, as a superfamily. Our results can be considered partially consistent with their proposal. A standard assessment method, reporting, and clinical management of PRAIO should be discussed urgently.

Together with the results of the cognitive survey and clinicopathological analysis, PRAIO can be assessed as a distinct tumor entity in future. Also, pathological characters of PRAIO determined in this study can serve as a baseline to establish the criteria of PRAIO. Establishment of PRAIO as a new entity will contribute to more standardized patient management and data set establishment for both PDAC and extrahepatic bile tract cancer.

Our study had several limitations. First, the PRAIO cases identified in our study may be indistinguishable from collision tumors involving multiple cancers arising from the biliary tract and the pancreas. Second, the method of preparation of tissue specimens is different between eastern and western countries. Third, it was difficult to perform multivariate analysis considering all the eight pathological factors because the number of patients with an unknown primary tumor was small. The distinction from collision tumors can be reconsidered and analyzed by collecting a larger sample according to our minimum PRAIO criteria. Although we had few cases of PRAIO and it was difficult to perform multivariate analysis considering all eight factors, building a scoring system can be considered with the features to distinguish PRAIO more appropriately in further studies.

In conclusion, our study showed the clinicopathological features of PRAIO and reported that the concept of PRAIO could be shared among pathologists. Based on the substantial frequency, unique clinicopathological features, and complexity involved in determining the primary tumor site, we recommend that PRAIO should be assessed as a distinct tumor entity. This will provide a more standardized data set for PDAC, AmpC, and DBDC, and will lead to the development of appropriate therapies for periampullary adenocarcinomas.

## DISCLOSURE

The authors have no conflicts of interest directly relevant to the content of this article.

## AUTHOR CONTRIBUTION

Ryuji Komine and Motohiro Kojima designed and conceived this study. Ryuji Komine: Patients enrollment, data acquisition, patient evaluation, and article preparation. Motohiro Kojima: Project administration, patient enrollment, data acquisition, and article review. Genichiro Ishii, Masashi Kudo, Motokazu Sugimoto, Shin Kobayashi, Shinichiro Takahashi, Masaru Konishi, Tatsushi Kobayashi, and Naoto Gotohda: Patient evaluation and article review. Tetsuo Akimoto: Article review. Ayumi Murakami, Motoko Sasaki, Mariko Tanaka, Akiko Matsuzaki, Nobuyuki Ohike, Katsunori Uchida, Tomoko Sugiyama, Kenichi Hirabayashi, Takuma Tajiri, Kazuyuki Ishida, Keita Kai, Yuko Omori, Kenji Notohara, Hiroshi Yamaguchi, Yoko Matsuda, Yoshiki Naito, Yuki Fukumura, Yoshihiro Hamada, Yumi Mihara, and Yohei Masugi: Participation in questionnaire survey and article review. Kenichi Harada, Noriyoshi Fukushima, and Toru Furukawa: Project administration and supervision, and article review. All authors read and approved the final manuscript.

## ETHICAL APPROVAL

The study conforms to the provisions of the Declaration of Helsinki and was approved by the hospital ethics committee (approval number: 2017–483).

## Supporting information

Fig S1

Table S1

## References

[1] Bosman FT , Carneiro F , Hruban RH , Theise ND . WHO Classification of Tumours of the Digestive System, 4th edn. Lyon: IARC Press; 2010.

[2] Amin MB , Edge S , Greene F , et al. AJCC Cancer Staging Manual, 8th edn. New York, NY: Springer; 2017.

[3] Rahib L , Smith BD , Aizenberg R , Rosenzweig AB , Fleshman JM , Matrisian LM . Projecting cancer incidence and deaths to 2030: the unexpected burden of thyroid, liver, and pancreas cancers in the United States. Cancer Res. 2014;74:2913‐2921. 24840647 10.1158/0008-5472.CAN-14-0155

[4] Fernández Moro C , Fernandez‐Woodbridge A , Alistair D'souza M , et al. Immunohistochemical typing of adenocarcinomas of the pancreatobiliary system improves diagnosis and prognostic stratification. PLoS One. 2016;11:e0166067. 27829047 10.1371/journal.pone.0166067 PMC5102456

[5] Siegel RL , Miller KD , Jemal A . Cancer statistics, 2018. CA Cancer J Clin. 2018;68:7‐30. 29313949 10.3322/caac.21442

[6] Randi G , Malvezzi M , Levi F , et al. Epidemiology of biliary tract cancers: an update. Ann Oncol. 2009;20:146‐159. 18667395 10.1093/annonc/mdn533

[7] Katanoda K , Matsuda T , Matsuda A , et al. An updated report of the trends in cancer incidence and mortality in Japan. Jpn J Clin Oncol. 2013;43:492‐507. 23493744 10.1093/jjco/hyt038

[8] Kojima M , Sudo H , Kawauchi J , et al. MicroRNA markers for the diagnosis of pancreatic and biliary‐tract cancers. PLoS One. 2015;10:e0118220. 25706130 10.1371/journal.pone.0118220 PMC4338196

[9] Chun YS , Pawlik TM , Vauthey JN . 8th edition of the AJCC cancer staging manual: Pancreas and hepatobiliary cancers. Ann Surg Oncol. 2018;25:845‐847. 28752469 10.1245/s10434-017-6025-x

[10] Bledsoe JR , Shinagare SA , Deshpande V . Difficult diagnostic problems in pancreatobiliary neoplasia. Arch Pathol Lab Med. 2015;139:848‐857. 26125425 10.5858/arpa.2014-0205-RA

[11] Adsay NV , Bagci P , Tajiri T , et al. Pathologic staging of pancreatic, ampullary, biliary, and gallbladder cancers: pitfalls and practice limitations of the current AJCC/UICC TNM staging system and opportunities for improvement. Semin Diagn Pathol. 2012;29:127‐141. 23062420 10.1053/j.semdp.2012.08.010

[12] Yoshida N , Esaki M , Kishi Y , et al. Bile duct carcinoma involving the common channel associated with pancreaticobiliary maljunction shows an extension pattern similar to ductal carcinoma of the pancreas. Pathol Int. 2013;63:415‐418. 23957917 10.1111/pin.12085

[13] Adsay V , Logani S , Sarkar F , Crissman J , Vaitkevicius V . Foamy gland pattern of pancreatic ductal adenocarcinoma: a deceptively benign‐appearing variant. Am J Surg Pathol. 2000;24:493‐504. 10757396 10.1097/00000478-200004000-00003

[14] Albores‐Saavedra J , Delgado R , Henson DE . Well‐differentiated adenocarcinoma, gastric foveolar type, of the extrahepatic bile ducts: a previously unrecognized and distinctive morphologic variant of bile duct carcinoma. Ann Diagn Pathol. 1999;3:75‐80. 10196386 10.1016/s1092-9134(99)80033-1

[15] Hruban RH , Adsay NV , Albores–Saavedra J , et al. Pancreatic intraepithelial neoplasia: a new nomenclature and classification system for pancreatic duct lesion. Am J Surg Pathol. 2001;25:579‐586. 11342768 10.1097/00000478-200105000-00003

[16] Maitra A , Adsay NV , Argani P , et al. Multicomponent analysis of the pancreatic adenocarcinoma progression model using a pancreatic intraepithelial neoplasia tissue microarray. Mod Pathol. 2003;16:902‐912. 13679454 10.1097/01.MP.0000086072.56290.FB

[17] Zen Y , Aishima S , Ajioka Y , et al. Proposal of histological criteria for intraepithelial atypical / proliferative biliary epithelial lesions of the bile duct in hepatolithiasis with respect to cholangiocarcinoma: preliminary report based on interobserver agreement. Pathol Int. 2005;55:180‐188. 15826244 10.1111/j.1440-1827.2005.01816.x

[18] Sato Y , Sasaki M , Harada K , et al. Pathological diagnosis of flat epithelial lesions of the biliary tract with emphasis on biliary intraepithelial neoplasia. J Gastroenterol. 2014;49:64‐72. 23616173 10.1007/s00535-013-0810-5

[19] Gonzalez RS , Bagci P , Basturk O , et al. Intrapancreatic distal common bile duct carcinoma: analysis, staging considerations, and comparison with pancreatic ductal and ampullary adenocarcinomas. Mod Pathol. 2016;29:1358‐1369. 27469329 10.1038/modpathol.2016.125 PMC5598556

[20] Vaccaro V , Sperduti I , Milella M . FOLFIRINOX versus gemcitabine for metastatic pancreatic cancer. N Engl J Med. 2011;365:768‐769. 21864184 10.1056/NEJMc1107627

[21] Klaiber U , Leonhardt CS , Strobel O , Tjaden C , Hackert T , Neoptolemos JP . Neoadjuvant and adjuvant chemotherapy in pancreatic cancer. Langenbecks Arch Surg. 2018;403:917‐932. 30397779 10.1007/s00423-018-1724-8

[22] Wang G , Wang Q , Fan X , Ding L , Dong L . The significance of adjuvant therapy for extrahepatic cholangiocarcinoma after surgery. Cancer Manag Res. 2019;11:10871‐10882. 31920396 10.2147/CMAR.S224583 PMC6941596

[23] Schmuck RB , de Carvalho‐Fischer CV , Neumann C , Pratschke J , Bahra M . Distal bile duct carcinomas and pancreatic ductal adenocarcinomas: postulating a common tumor entity. Cancer Med. 2016;5:88‐99. 26645826 10.1002/cam4.566 PMC4708893

