# Supplementary figures and images for: Recognition and pathological features of periampullary region adenocarcinoma with an indeterminable origin
Source: Cancer Med. 2021 May 19;10(11):3499–510. doi: 10.1002/cam4.3809 (PMC8178491; doi:10.1002/cam4.3809)

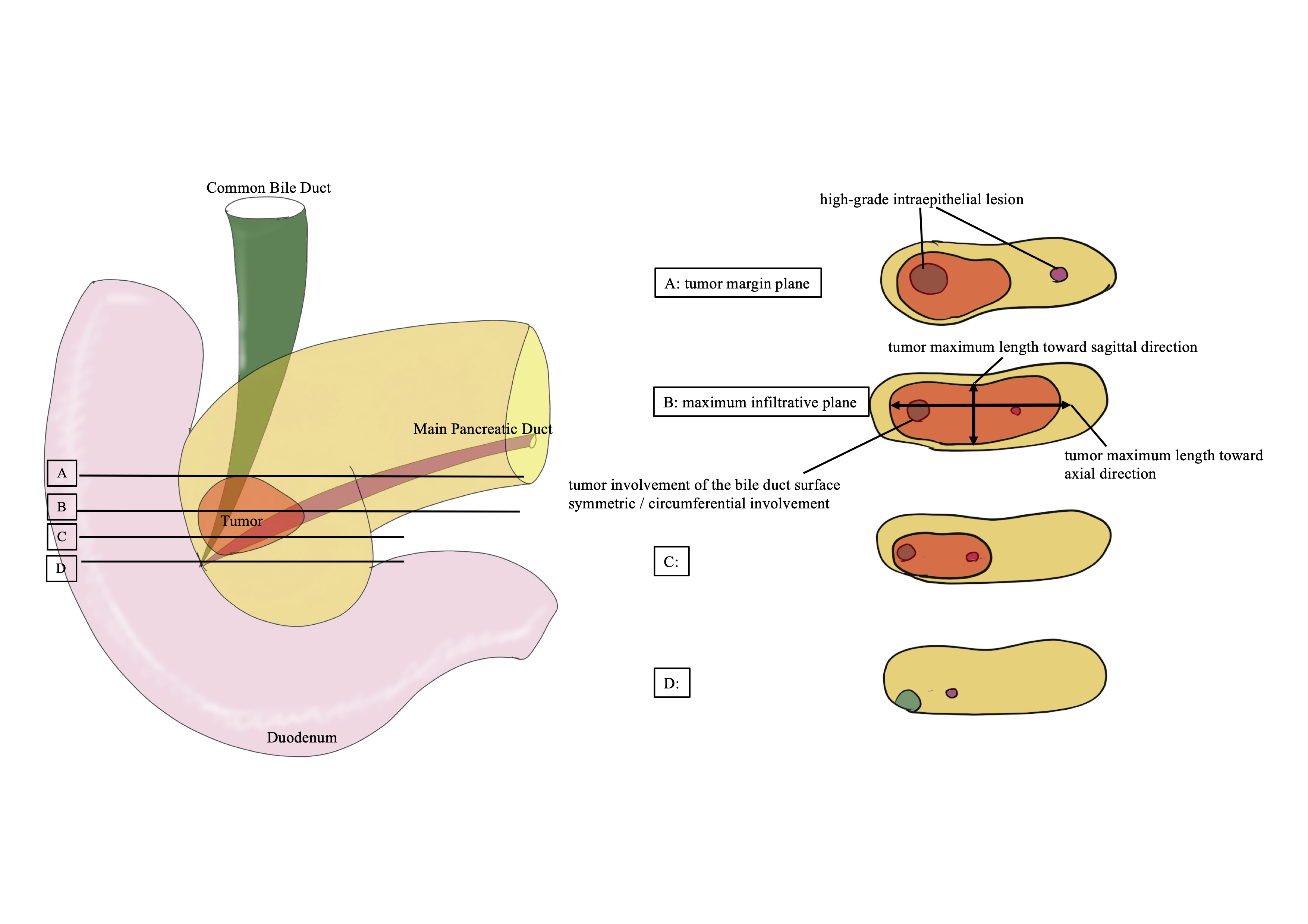

Supplement: Supplementary file 1 — Fig S1 [file CAM4-10-3499-s002.jpg]
