# Supplementary material for: Recognition and pathological features of periampullary region adenocarcinoma with an indeterminable origin
Source: Cancer Med. 2021 May 19;10(11):3499–510. doi: 10.1002/cam4.3809 (PMC8178491; doi:10.1002/cam4.3809)
Supplement: Supplementary file 2 — Table S1 [file CAM4-10-3499-s001.docx]

Table S1: Questionnaire for cognitive survey

| No. | Question | Alternatives |
| --- | --- | --- |
| 1 | Period of experience as a pathologist. | Please answer years |
| 2 | Have you ever experienced cases where the tumor primary could not be determined within periampullary region?  If you answered "yes" in question 2, please also answer Question No.2A and 2B. | 1. Yes 2. No |
| 2A | How frequent have you experienced? | 1. One in 10 cases 2. One in 25 cases 3. One in 50 cases 4. One in 100 cases 5. More than above |
| 2B | How do you report such cases? | 1. As PDAC 2. As BTC 3. Both PDAC and BTC (List both TNM staging) |
| 3 | Which findings do you think important to determine tumor primary?  (multiple select from following). | 1. macroscopic findings 2. occupation area of microscopically infiltration 3. existence of normal epithelium or existing structures. 4. continuity of epithelium and infiltration. 5. infiltration along long axis direction of the CBD or MPD. 6. carcinoma in situ or cancerization at the tumor margin. 7. other helpful histological findings. 8. clinical information. 9. other. |
| 4 | Which is the most important finding to determine the tumor primary? | Select from alternatives of Question No.3 |
| 5 | Have you ever experienced cases in which clinical and pathological diagnosis of tumor primary did not met? | 1. Yes 2. No |
| 6 | Which finings do you think important to decide "tumor main location"?  (multiple select from following). | 1. macroscopic findings 2. wide area of microscopically infiltration 3. existence of infiltration along long axis direction of the CBD or MPD 4. existence of infiltration into around tissue 5. other |
| 6A | Which is the most important finding to decide "tumor main location"? | Select from alternatives of Question No.6 |
| 7 | Which findings do you think useful to determine "tumor main location" in the tumors evenly involving the bile duct and the pancreas, macroscopically?  (multiple select from following) | 1. macroscopic findings (decide only by macroscopic findings) 2. wide area of microscopically infiltration (decide by the more widely infiltration area) 3. presence of carcinoma in situ, cancerization or precursor findings around the tumor 4. clinical information 5. other |
| 7A | Which is the most important finding to decide "tumor main location" in Q7 case? | Select from alternatives of Question No.7 |
| 7B | Which findings do you think important to determine "tumor main location" in tumor evenly involving the bile duct and the pancreas macro and microscopically?  (multiple select from following) | 1. the size of microscopical infiltration area (Wider area is regarded as main location) 2. presence of normal epithelium or existing structures (remaining area is considered not as main location) 3. presence of continuity of epithelium and infiltration (continuity area is considered as main location) 4. presence of carcinoma in situ or precursor lesion like PanIN/BilIN at the tumor margin 5. presence of infiltration along long axis direction of the CBD or MPD 6. presence of cancerization, 7. clinical information 8. other |
| 7C | Which is the most important finding to determine "tumor main location" in Q7B case? | Select from alternatives of Question No.7B |
| 8 | Do you feel a limitation to determine the tumor origin only from histological findings? | 1. Yes 2. No |
